# Multi-dimensional designer catalysts for negative emissions science (NES): bridging the gap between synthesis, simulations, and analysis

**DOI:** 10.1016/j.isci.2021.103700

**Published:** 2021-12-27

**Authors:** Caleb M. Hill, Jose L. Mendoza-Cortes, Jesús M. Velázquez, Luisa Whittaker-Brooks

**Affiliations:** 1 Department of Chemistry, University of Wyoming, 1000 E University Avenue, Laramie, WY 82071, USA; 2 Department of Chemical Engineering & Materials Science, Michigan State University, East Lansing, MI 48824, USA; 3 Department of Chemistry, University of California, Davis, Davis, CA 95616, USA; 4 Department of Chemistry, University of Utah, Salt Lake City, UT 84112, USA

**Keywords:** Catalysis, Electrochemistry, Materials science, Materials synthesis, Computational materials science

## Abstract

Negative emissions technologies will play a critical role in limiting global warming to sustainable levels. Electrocatalytic and/or photocatalytic CO_2_ reduction will likely play an important role in this field moving forward, but efficient, selective catalyst materials are needed to enable the widespread adoption of these processes. The rational design of such materials is highly challenging, however, due to the complexity of the reactions involved as well as the large number of structural variables which can influence behavior at heterogeneous interfaces. Currently, there is a significant disconnect between the complexity of materials systems that can be handled experimentally and those that can be modeled theoretically with appropriate rigor and bridging these gaps would greatly accelerate advancements in the field of Negative Emissions Science (NES). Here, we present a perspective on how these gaps between materials design/synthesis, characterization, and theory can be resolved, enabling the rational development of improved materials for CO_2_ conversion and other NES applications.

## Graphical abstract

**Figure:**
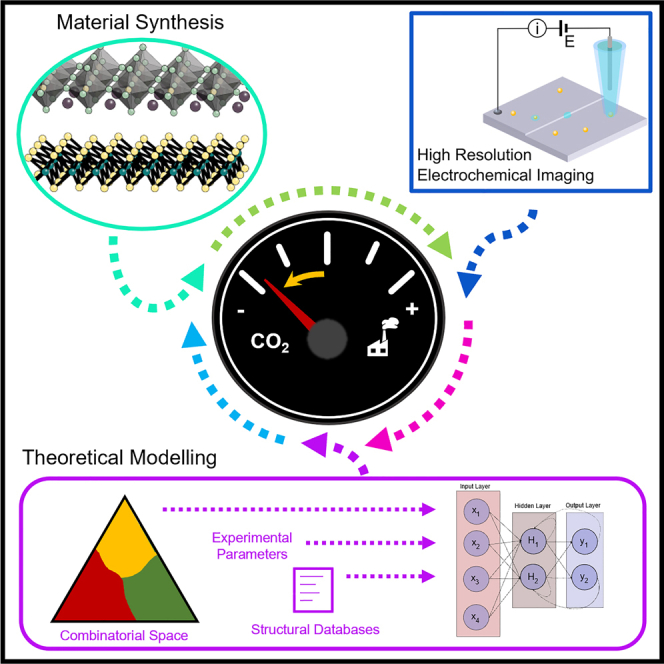

## Introduction

The wide-scale adoption of negative emissions technologies will play a critical role in limiting global warming to sustainable levels. A significant amount of research has focused on developing technologies that would remove and sequester carbon from the atmosphere (Jiang et al., 2020b, National Academies of Sciences, Engineering, and Medicine, 2019). Anthropogenic sources such as fossil fuel consumption, agricultural activities, and cement production have been identified as massive producers of atmospheric CO_2_ (Minx et al., 2018; Fuss et al., 2018). Given the imminent risk and damage associated with the uncontrollable increase of CO_2_ in the atmosphere, several countries have made it a priority to reduce its concentration by 80%–100% by 2050 (Meckling and Biber, 2021;Van Vuuren et al., 2018). When combined with direct air capture technologies, the catalytic conversion of CO_2_ into high-value products may offer an economically viable option for atmospheric CO_2_ remediation, a central goal in negative emissions science (NES). The catalytic reduction of CO_2_ can be performed either via an electrocatalytic or a photocatalytic process. In electrocatalytic CO_2_ conversion systems, electrical energy is utilized to drive CO_2_ reduction reactions (CO_2_RRs) to form useful products at the surface of a cathode in an electrochemical cell. A hallmark example is the production of formic acid from CO_2_, which can be described via the following reaction in basic media:

Equation 1 $$\begin{array}{l} {\text{CO}}_{2}+{\text{H}}_{2}\text{O}+2e^{-}\to {\text{HCOO}}^{-}+{\text{OH}}^{-}\;{\text{E}}^{0^{\prime }}=-0.43\;V\;vs.\;SHE\;\left(\text{pH}=7\right) \end{array}$$

**Figure 1:**
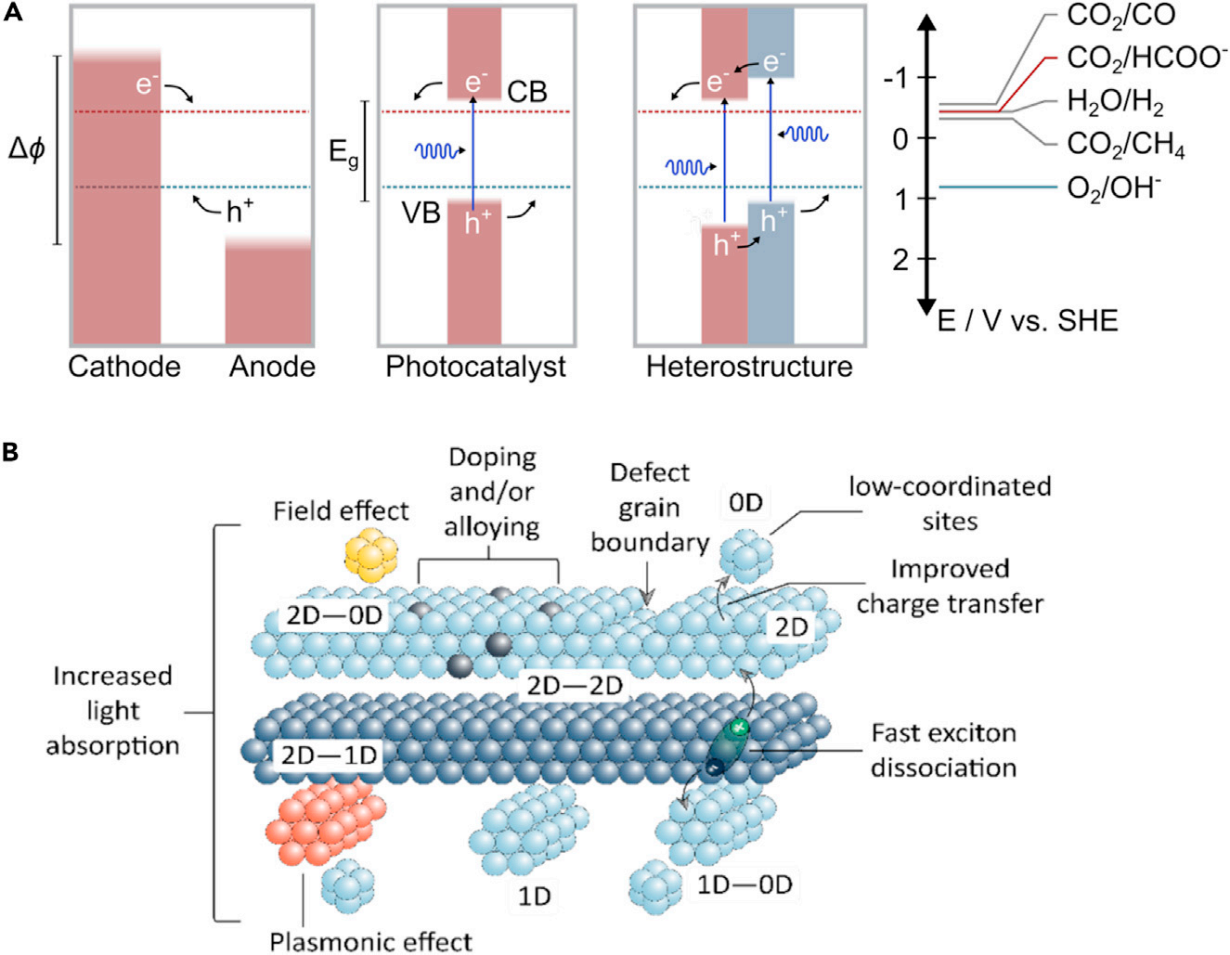
Catalytic systems for CO_2_ reduction (A) Band alignments involved in different schemes for CO_2_ reduction. Electrocatalytic CO_2_ reduction reactions can be driven under dark conditions by applying an electrical bias (Δφ) between two electrodes in an electrochemical cell. Alternatively, these reactions can be driven by energetic photogenerated carriers in the conduction band (CB) and valence band (VB) of a semiconductor photocatalyst, or heterostructures thereof. (B) An illustration of the various structural parameters which can influence the performance of a heterostructured photocatalyst system. Light absorption and carrier transport can be controlled by coupling semiconductor and/or metal structures of varying dimensionality, while reaction kinetics at the surface can influenced by atomic-scale motifs at the interface, such as dopants, vacancies, or other crystallographic defects. Adapted with permission from *Nat. Rev. Chem.***2018**, *2*, 0105. Copyright © 2018. Springer Nature (Voiry et al., 2018).

The reduction of CO_2_ into value-added molecules and fuels is a thermodynamically unfavorable process due to the high stability of the C=O bond (750 kJ mol^−1^ as compared with 411 kJ mol^−1^ for a C-H bond, for example) (Wang et al., 2019). Given the large amount of energy needed to break the C=O bond, many research efforts have focused on developing solar-driven, or photocatalytic, processes that would facilitate the reduction of CO_2_ (Vogt et al., 2019). However, developing efficient photocatalytic reduction processes is a non-trivial task given that CO_2_ is optically inert at solar photon energies (UV and visible wavelengths). Photocatalytic CO_2_ reduction systems must therefore utilize light-absorbing materials, commonly semiconductors, which can generate the excited carriers necessary to drive these reactions. For CO_2_ reduction, a photocatalyst material must possess a conduction band which is higher in energy (more negative) than the reduction potential for the relevant reaction. Conversely, a material must possess a low-lying valence band in order to drive a complementary oxidation reaction (e.g., oxygen evolution) (Figure 1A).(Voiry et al., 2018) Heterostructures between multiple semiconducting materials can enhance performance due to built-in electric fields which can drive the transport of carriers anisotropically, minimizing losses due to carrier recombination and directing transport to active sites for each half-reaction. Despite the great appeal of these photocatalytic CO_2_ conversion schemes, it has proven difficult to develop systems with the efficiency, selectivity, and low cost necessary to find practical, large-scale application.

A central challenge for the development of CO_2_ conversion systems has been the catalyst materials where CO_2_ reduction reactions occur. The surface of a catalyst material provides the active sites necessary to bind unstable reaction intermediates involved in complex reactions. For example, the reduction of CO_2_ to HCOO^−^ in Equation 1 can be considered a two-step process where CO_2_ first undergoes a proton-coupled electron transfer event to produce an HCOO∗ intermediate bound to the catalyst surface (Yoo et al., 2016; Birdja et al., 2019). The local structure of the catalyst surface dictates the relative stability of intermediates involved in different reaction pathways, and thus plays a key role in determining both product selectivity and energetic efficiency. In photocatalytic systems, the electronic structure of the material is also critical, as it dictates how efficiently light is converted into energetic carriers and how these carriers are transported to key catalytic sites. Accordingly, improved catalyst materials, including photocatalysts, will be key to the development of practical systems for NES applications.

Rationally designing better catalyst materials will require a detailed understanding of how different structural parameters influence the stabilization of key reaction intermediates, how excited carriers are generated, and how these carriers are transported. Unfortunately, the heterogeneous nature of practical catalyst systems makes it fundamentally challenging to generate detailed structural information. While a catalyst material may possess a well-defined bulk composition and crystal structure, surfaces exhibit a wide array of different structural motifs (edges/steps, adatoms, vacancies, substitutional dopants, etc.) which possess distinctly different chemical and optoelectronic properties. This complex situation even assumes the surface does not change over the course of the reaction. And while the synthesis of more elaborate, heterostructured catalyst systems may offer important advantages, they also add yet another layer of complexity. Further complicating matters is the disconnect between the complexity of these macroscopic surfaces which are studied using traditional experimental techniques and the possible atomistic models which are computationally achievable using current theoretical methods.

Bridging the gaps between synthesis, experiment, and theory will be critical to enable the true rational design of materials for NES applications but will require the concerted effort of scientists working in each of these areas. Materials chemists must work to synthesize materials with a controlled variety of structural motifs which influence performance, measurement scientists must develop analytical techniques which can probe well-defined motifs across different length scales within these materials systems, and theoreticians must work to develop novel computational and modeling methods which can rationalize the behavior of these complex systems and predict synthesizable materials to target. In this perspective, we explore each component of this problem using heterostructured metal chalcogenides as a case study within the context of materials for electro- and photocatalytic CO_2_ reduction. We hypothesize that this model system, in which we can vary different structural parameters such as layer arrangement, heterostructure formation with other multidimensional components, or defect/dopant concentration within surfaces (Figure 1B), will advance our fundamental understanding of how to design better catalyst materials for CO_2_ reduction. Promising strategies to advance each area are described, inspiring an outlook of how synthesis, theory, and analysis may together enable the rational development of improved materials for CO_2_ conversion and other NES applications.

## Metal chalcogenides as electrocatalysts for CO_2_ reduction

Dimensionally controlled chalcogenides may serve as tunable building blocks for cross-compositional and cross-dimensional heterostructuring toward the synergistic improvement of material properties and surface reactivity(Perryman and Velázquez, 2021; Giuffredi et al., 2021). This is critical to draw connections between dimensionality, morphology, composition, and reaction trajectories (e.g. CO_2_ reduction to higher alcohols and hydrocarbons). CO_2_ reduction represents a vast field of research that has recently expanded into the realm of metal chalcogenides (Perryman et al., 2020b; Abbasi et al., 2017; Asadi et al., 2016). Much promise has been attributed to singular metal-chalcogenide-based electrocatalysts for CO_2_ reduction due to their ability to break pernicious intermediate scaling relations that plague non-copper monometallic surfaces (Chan et al., 2014; Hong et al., 2016). However, the majority of work in this area to date has been restricted to theoretical explorations of adsorbate binding energies on 2D-vdW structures with and without a ternary dopant, a dangling bond at an edge site (Hong et al., 2016) or vacancies at a basal plane(Le et al., 2014). To the best of our knowledge, few publications have established sufficient control over synthetic processing for 2D materials to achieve these surface motifs and compositional anomalies that are predicted to drive (in-operando) favorable and selective CO_2_ conversion.

Among the bottom-up methods that can provide the necessary dimensional and topological control, metal-organic chemical vapor deposition (MOCVD) of large-area and defect-controlled vdW monolayers and multilayers offers an attractive route for the synthesis of model systems over which we can pursue atomic-level surface science in conjunction with electrochemistry (Ishihara et al., 2018). Establishing a reliable protocol for synthesizing >1cm^2^ coverage of MoS_2_ having well-defined S vacancy distributions, for example, would allow for the confirmation of ideas that have been previously discussed regarding the intrinsic difference in CO_2_ reduction reactivity at a row of S vacancies versus a patch of S vacancies (Le et al., 2014). In successfully synthesizing these model systems, we can implement widely accessible methodologies that will allow us to glean insights into the role of structural defects on the binding geometry of reaction intermediates that exist on vdW surfaces at a timescale such that transient spectroscopy is sufficient for analysis. Moreover, the well-defined and atomically smooth nature of these model systems obviates the need for extensive post-synthesis processing that would be required prior to performing surface-sensitive and chemically-specific characterization techniques like grazing incidence X-ray absorption spectroscopy (XAS). In conjunction with traditional electrochemical quantification of CO_2_ reduction reactivity, these in-depth experimental investigations of vdW structure–reactivity relationships constitute a much-heralded avenue of interdisciplinary research in the fields of chemistry, physics, and materials science both at the theoretical and experimental level.

**Figure 2:**
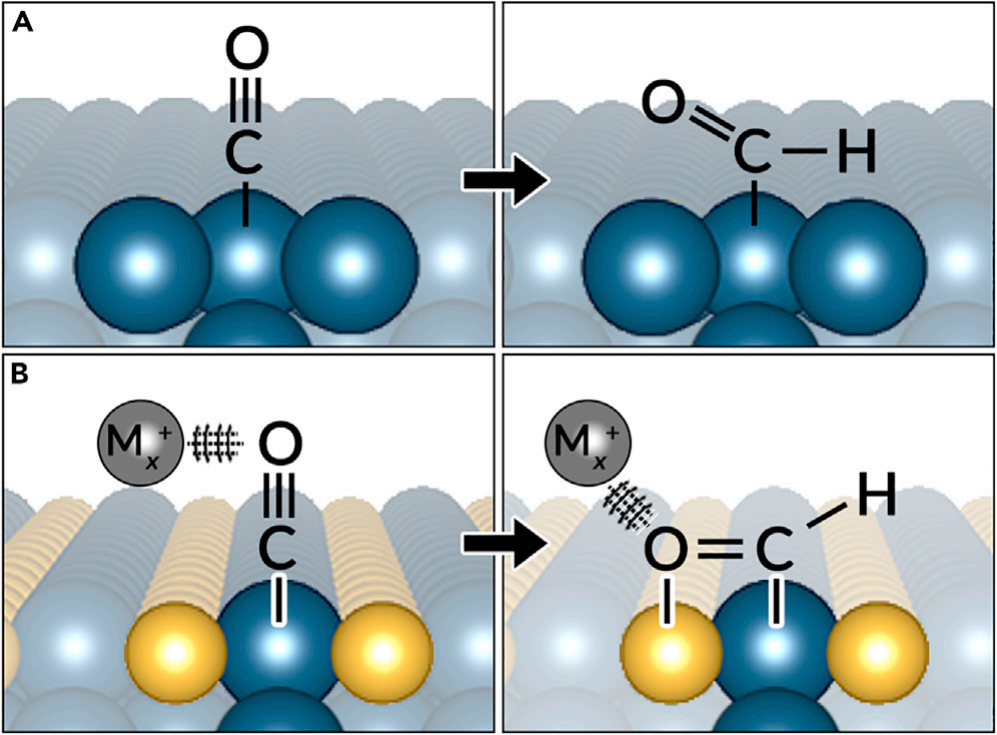
Metal chalcogenides as catalysts for CO_2_ reduction (A) CO hydrogenation over a purely metallic surface (A) compared to a metal chalcogenide surface. (B) Coulombic stabilization by M_x_cation (*gray*) of anionic intermediate domains is included to show secondary interaction for selectivity.

To advance our fundamental understanding of photocatalytic CO_2_ reduction, we must have a testbed where we can vary the different variables in the reaction. We have this potential testbed in 2D cross-dimensional heterostructures (*vide infra*). However, we must establish surface structure–reactivity relationships taking into account the surface and bulk composition which can help accelerate discovery and design (Perryman et al., 2020a; Ortiz-Rodríguez et al., 2020). Where 2D vdW structures are posited to afford bidentate coordination environments (Figure 1A) for catalytically important intermediate species in the CO2RR as an alternative to potentially less favorable monodentate structures (Figure 1B), it has been shown that the ternary composition of 3D chalcogenides like Chevrel phases affords a similar level of control over adsorbate binding (Liu et al., 2010; Liu and Liu, 2015). This stems from the intrinsic bifunctional nature of multinarychalcogenides wherein metal sites and chalcogen sites yield inherently different affinities for carbon and oxygen domains—a phenomenon that is amplified by the inclusion of a ternary species, often one that is cationic in nature, that can either directly participate in adsorbate binding or that can indirectly stabilize intermediates by attenuating their observable electric field at an electrode surface. This latter effect (Figure 2B, right side) has been coined as the “ensemble effect” and represents an extensive research space for elucidating the fundamentals of CO_2_ reduction chemistry on dimensionally and compositionally controlled surfaces(Liu and Nørskov, 2001).

Beyond simply controlling local coordination via compositional modification in flexible 3D chalcogenide frameworks, it is also understood—albeit underexplored—that even morphological changes in 3D systems can introduce profound changes in locally observed electric fields for CO_2_ reduction intermediates (Gao et al., 2012, 2020). This strongly motivates the exploration of additional synthetic techniques that will enable control over particle morphology on well-defined chalcogenide compositions (Perryman et al., 2020a), similar to the established hydrothermal and solvothermal routes that have been employed for metal oxide nanoparticle catalysts for thermal CO_2_ reduction (Jiang et al., 2020a; Liang et al., 2014).

**Figure 3:**
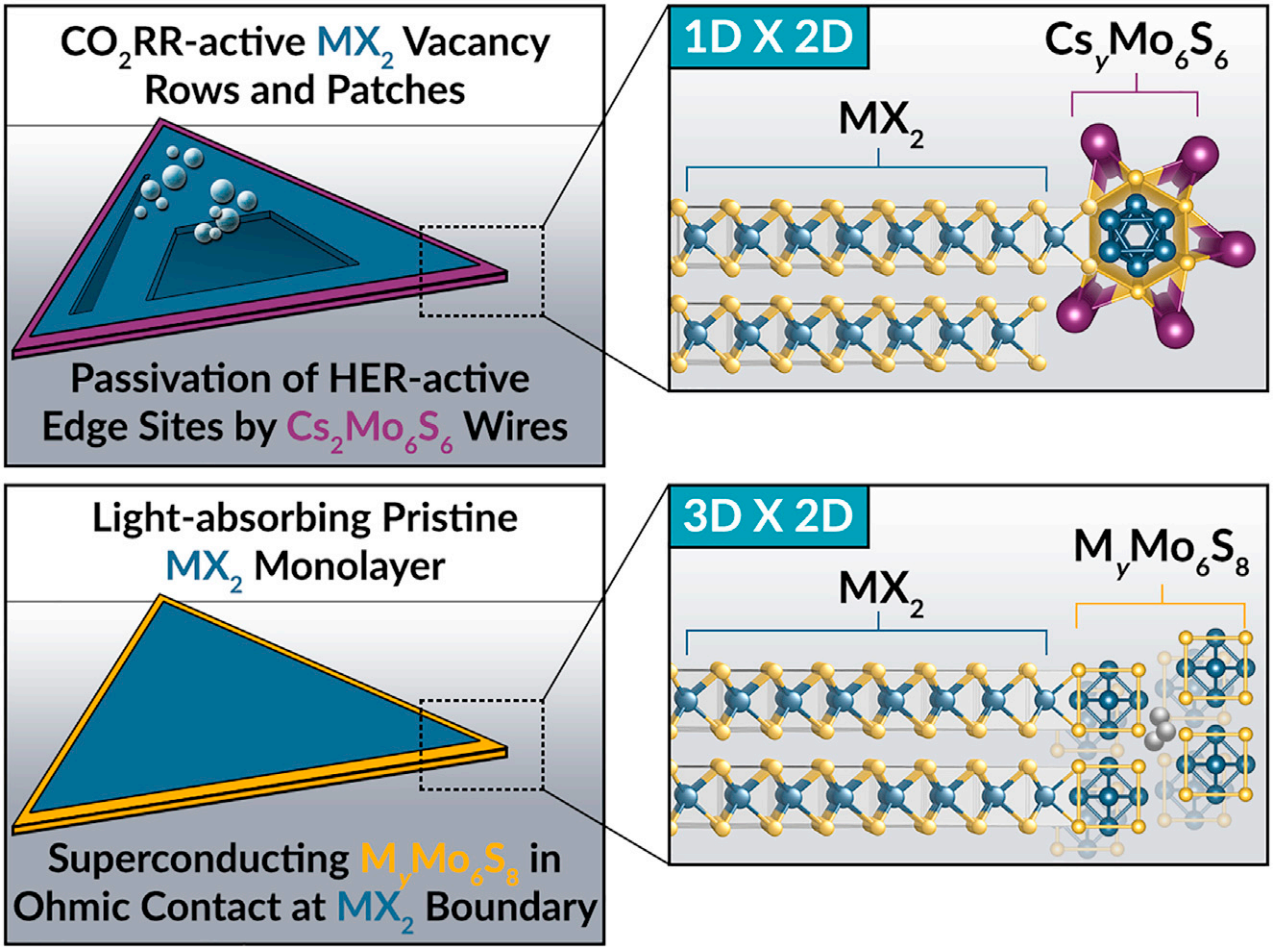
Cross-dimensional heterostructures for CO_2_ reduction Cross-dimensional chalcogenide heterostructures can include multifunctional junctions between catalytically activated MX_2_ surfaces and HER-suppressive psuedo-chevrel-phase (PCP) surfaces (top) and between pristine light-absorbing MX_2_ surfaces and CO_2_ electroreduction mediating PCPs and Chevrel phases (CPs) (bottom).

Understanding structure–reactivity relationships in individual 2D and 3D chalcogenides is an incredibly open area of study owing to our current lack of atomistic understanding of the interplay between CO_2_ reduction active site environment, electronic structure, and adsorbate activation. Perhaps even more so, our ability to interface chalcogenide materials across these two dimensionalities, when coupled with the interesting potential additive CO_2_RR reactivity at 2D/3D heterostructured interfaces (Figure 3), makes investigating combinations of 2D structures with 3D structures particularly intriguing. We have posited the interesting nature of cross-dimensional heterostructures on the grounds of their fast exciton transport across interfaces, although it is also worth exploring the effect on CO_2_ reduction reactivity at interfaces wherein lattice mismatch-induced electrical impedance could serve to slow exciton transport and potentially act to cool 2D vdW-derived hot carriers in a photoelectrochemical system (Furchi et al., 2018; Wang et al., 2021). Whether this process is radiative or non-radiative and whether that enables matching of exciton generation timescales with adsorption and intermediate activation timescales remains to be seen. Hence achieving interfaces between structures with particularly matched phonon energies and electrical conductivities may enable fine control over the directionality and localization of energetically significant charge carriers for electrocatalytic redox chemistry.

## Mixed dimensional and hierarchical photocatalysis for CO_2_ conversion

Given that CO_2_ is optically inert at high photon energies, several research strategies have resorted to the development of low-dimensional photocatalysts with a suitable bandgap and an appropriate energy band alignment where the conduction band (CB) minimum must be negative with respect to the redox potential of CO_2_ (Figure 1A). 2D photocatalysts (aka 2-periodic) based on metal chalcogenides (e.g. MoS_2_, SnS_2_, WSe_2_, Bi_2_S_3_, Chevrel phases, to mention a few) (Rahman et al., 2016; Degrauw et al., 2017; Ortiz-Rodríguez et al., 2020; Perryman et al., 2020a, 2020b; Perryman and Velázquez, 2021; Hill and Hill, 2019, 2021; Strange et al., 2020; Hill et al., 2020; Tolbert and Hill, 2021) and 2D metal halide perovskites (Amerling et al., 2020, 2021; Wu et al., 2021; Yuan et al., 2021; Pareja-Rivera et al., 2021) are well-suited materials for the reduction of CO_2_ due to their strong light-matter interactions. Although many low-dimensional photocatalysts have a high density of electronically active sites for CO_2_ binding, these same sites can act as recombination centers that detrimentally affect CO_2_ conversion efficiencies. These recombination centers can be significantly reduced in heterostructures with proper energy band alignment. 2-periodic materials often exhibit strong van der Waals (vdW) interactions which may enable the formation of mixed-dimensional heterostructures (0D-1D, 1D-1D, 1D-2D, 2D-2D) that can serve as effective catalysts for CO_2_ conversion to fuels.

**Figure 4:**
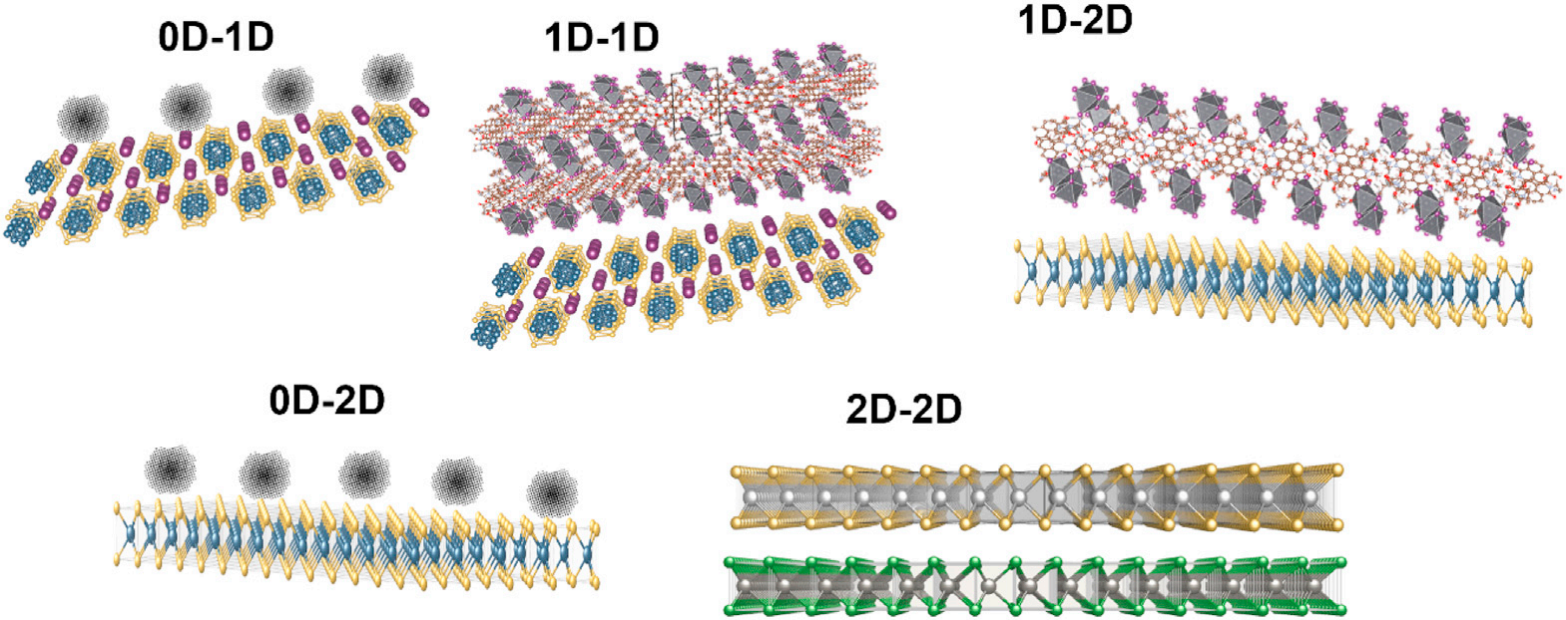
vdW heterostructures comprising mixed-dimensional moieties

Mixed-dimensional heterostructures (Figure 4) can be engineered to achieve hierarchical structures where charge transfer, exciton dissociation, and/or plasmon resonance may be modulated to afford highly active catalytic sites for CO_2_ binding (Voiry et al., 2018). Given the large number of individual building blocks available to form mixed-dimensional heterostructures, we believe it is important to develop theoretical and data science approaches that could streamline the identification of suitable compositions and dimensions to enable the assembly of defects in *p-n*heterojunctions. *p-n*heterojunctions based on vdW heterostructures are prone to defects such as dangling bonds, vacancies, crystal lattice mismatches, and interfacial defects which all need to be carefully characterized given their contributions to observable energy conversion efficiency. Several proof-of-concept mixed-dimensional vdW heterostructures have been demonstrated; however, there is a significant experimental gap when it comes to the fabrication of large-area and defect-controlled thin films with well-defined structures with few dangling bonds, low exciton binding energies, and fast charge transfer across the heterostructures as well as at the heterostructure/electrode interface. Here, theoretical studies are rare due to the large number of variables associated with the small concentration of defects present in these materials, however, some recent progress on modeling them has been made using density functional theory (DFT) and simple Hamiltonians (Pablo-Pedro et al., 2020). We believe that the rational design of large-area mixed-dimensional vdW heterostructured thin films may be achieved via MOCVD approaches (Olofinjana et al., 2019; Bhattacharyya et al., 2020). Recently, it was demonstrated that large-area, highly ordered thin film-based 2-periodic metal-organic frameworks (2D-MOFs) with large crystallite domains can be fabricated using CVD approaches (Ogle et al., 2021). This experimental approach can be combined with electronic structure calculations to design and predict several 2-periodic heterostructures and their properties for energy storage/conversion (Pakhira and Mendoza-Cortes, 2019). Thus, we believe that mixed-dimensional vdW heterostructures may be an excellent model system for understanding and designing CO_2_ reduction catalysts as well as designing defects and packing motifs, where we can combine experimental techniques (MOCVD fabrication), spectroscopy, theoretical approaches, and even data science due to the magnitude of data being generated and the different variables available for tuning the catalytic activity of materials.

In the same vein, specifics are still poorly understood on how CO_2_ is bound to surfaces containing different interaction modes (open metal sites, dangling bonds, point defects), the type of intermediate products that are formed, the influence of the electric field at the interface, and the origin of large CO_2_ reduction overpotentials due to the interplay between large thermodynamic barriers and kinetics, especially *in operando*. Similarly, little is known about the nature and number of active sites or how the host structure changes during the reaction. This is likely to occur in mixed-dimensional vdW heterostructures and other catalytic and light-absorbing materials. Thus, the rational design of heterostructures comprising less coordinated sites may further enhance their catalytic activity.

**Figure 5:**
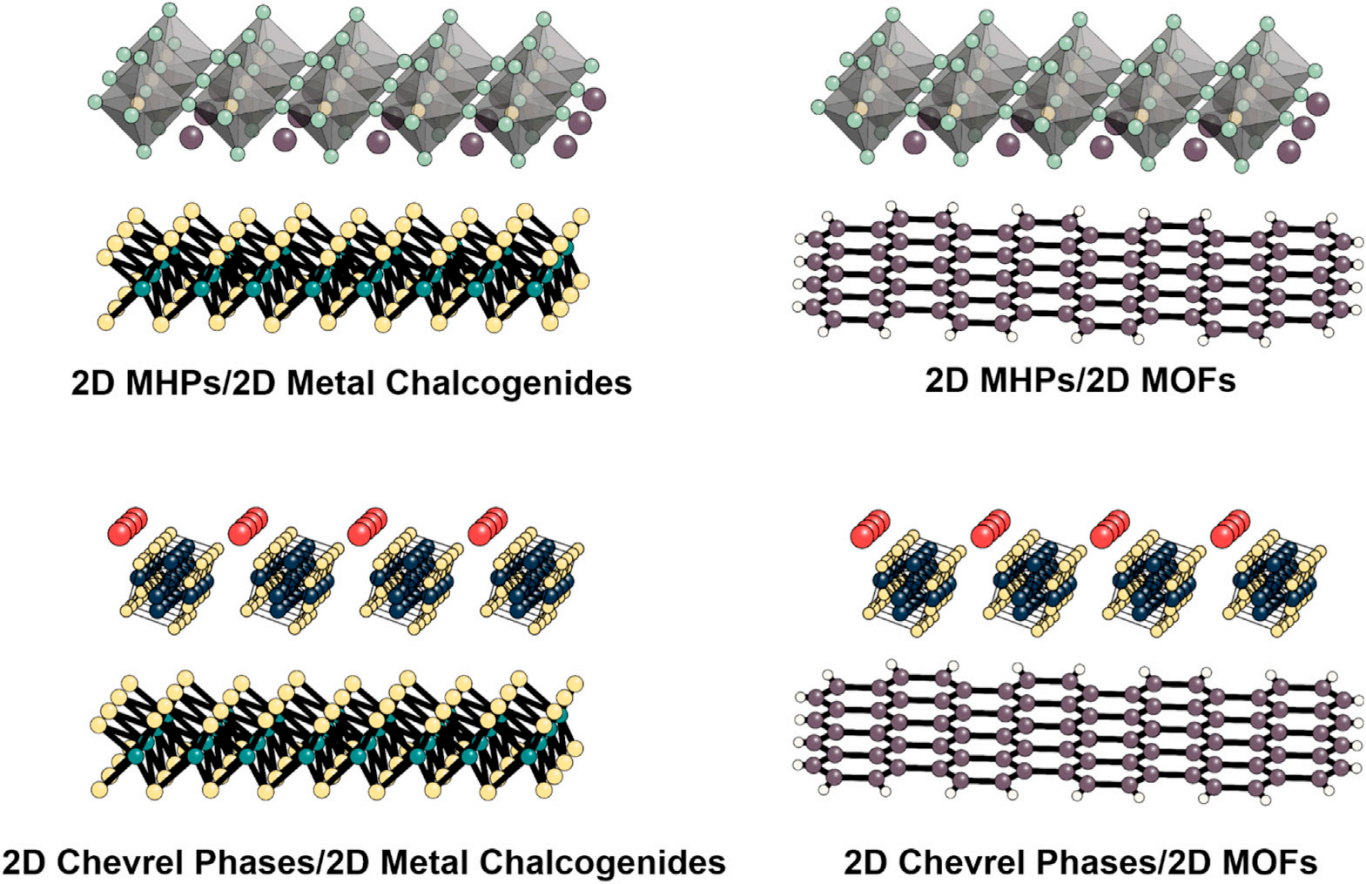
2D-2D vdW heterostructures for solar-driven CO_2_ reduction potential 2D-2D vdW heterostructure arrangements with optimal bandgap alignment for CO_2_ reduction from sunlight The top layer must have a bandgap of 1.8 eV while the bottom layer must have a bandgap of 1.2 eV for efficient tandem photocatalysis.

One of the main advantages offered by the development of mixed-dimensional vdW heterostructures is the potential of tuning the nature of the individual building blocks to achieve tandem configurations where high bandgap semiconductors could be replaced by two or more low bandgap semiconductors. Specifically, designing mixed-dimensional vdW heterostructures comprised of two semiconductors with bandgaps of 1.2 and 1.8 eV may yield more efficient photocatalysts for CO_2_ reduction due to higher solar absorption efficiencies and the generation of more favorable chemical interactions for complex CO_2_ reduction reactions. Figure 5 shows some potential 2D-2D arrangements that could be fabricated to fulfill the optimal bandgap requirement for tandem mixed-dimensional vdW heterostructures. A promising route to achieve an efficient tandem photocatalyst may be to combine 2D metal chalcogenides and 2DMOFs to tune the bandgap close to 1.2 eV to get protons from a water oxidation reaction while 2D metal halide perovskites (MHPs) and Chevrel phases could be used to tune the bandgap close to 1.8 eV to get enough energy to perform CO_2_ reduction reactions. In this approach, sunlight capture occurs in direct connection to the catalytic site, thus creating another form of artificial photosynthesis. The challenge will be to develop synthetic protocols that could potentially bridge MOCVD and solution-based protocols such as spin coating or Langmuir–Blodgett, in combination with analytical techniques. This, however, can be breached by synergistic interactions with theoretical/computational approach and data science, where theory can aim at design and mechanistic understanding, while data science can complement the multivariate analysis of experimental measurements and connections to computations.

Additionally, the 2D heterostructure arrangements should provide high current densities (>200 mA/cm^2^) given the high electron mobility (200–1,500 S cm^−1^) of the individual building blocks (Ogle et al., 2021; Ortiz-Rodríguez et al., 2020; Perryman et al., 2020a, 2020b; Perryman and Velázquez, 2021). An important research direction will be to model the catalytic activity of 2DMOFs, Chevrel phases, and 2DMHPs, their active sites, and selectivity toward reducing CO_2_ into high value-added molecules. Furthermore, as these mixed-dimensional vdW heterostructures have more surface-active sites, CO_2_ bubble formation becomes an important issue. Bubble formation, particularly at high applied potentials, may block active sites causing voltage instabilities that accelerate the degradation rate of the catalysts (Lee et al., 2020; Ren et al., 2020; Xing et al., 2021). To date, there have been no mechanistic studies on how CO_2_ bubble formation affects the current/voltage response of catalytic systems in terms of nucleation rate, lifetime, and internal pressure of the bubbles, especially for vdW heterostructures, nor has there been an overall study of degradation mechanisms for catalysts under large applied potentials or surrounded by high-energy excitons.

Finally, interesting suggestions have been proposed to develop hierarchical mixed-dimensional vdW heterostructures where their electro- and photocatalytic activity could be tuned through morphology, composition, defects, surface area overlap (e.g., 0D in contact with 2D), and strain engineering (Figure 1B). In these hierarchical structures, different 0D to 2D building blocks could be interfaced to enhance charge transfer, exciton dissociation, and plasmon resonance within one structure (Voiry et al., 2018). However, the assembly and understanding of the fundamental catalytic properties of these hierarchical structures may be extremely challenging. As such, experiments guided by predictive modeling and machine learning approaches may streamline the understanding and the design of complex catalytic systems toward higher efficiency, activity, and selectivity with prolonged stability under catalytic turnover.

## Advanced characterization of catalyst surfaces for CO_2_ reduction

Electrochemical systems are commonly characterized using a combination of bulk voltammetry and structural probes based on electron microscopy. Owing to the complexity of many electrode materials, information generated through traditional voltammetry contains contributions from millions or more distinct structural features distributed across a mm^2^-cm^2^ interface. It is thus impossible to conclusively identify the features which dictate the reactivity of the electrode surface, severely limiting the rational design of materials for a variety of electrochemical applications. More valuable insights into the behavior of electrode materials could undoubtedly be generated by evaluating the chemical behavior of individual, well-defined structural features.

While a variety of structural probes can provide information at the nm, or even atomic level, it is challenging to generate electrochemical information at comparable scales. This has long been a goal within the electrochemical community, and efforts have usually focused on employing different types of probes to locally interrogate extended electrochemical interfaces. A variety of optical detection schemes, such as plasmon resonance (Hill and Pan, 2013; Hill et al., 2015; Shan et al., 2012), fluorescence (Palacios et al., 2006; Xu et al., 2008; Sambur et al., 2016), or Raman scattering (Cortés et al., 2010; Zaleski et al., 2016) have been employed to locally interrogate electrochemical processes within macroscopic systems. Conventional optical systems can achieve diffraction-limited spatial resolution, but super-resolution optical techniques have been successfully applied to probe electrochemical reactions occurring within sub-100 nm domains (Mao et al., 2019). Unfortunately, most optical approaches are severely limited in terms of applicability, working only for a small set of materials or model reactions. Electrochemical microscopy techniques, such as scanning electrochemical microscopy (SECM), offer a more direct approach to probing the reactivity of well-defined structural motifs within an extended sample. SECM has been employed to locally probe catalytic behavior through the substrate generation-tip collection mode (Bard and Mirkin, 2012; Sun et al., 2014; Kim et al., 2016), where products generated locally at an entity of interest are detected at the tip electrode by driving a reverse reaction. This enables SECM to be used a tool for probing reactive intermediate species and thus providing insights into complex electrochemical reaction mechanisms, as demonstrated by Bard et al. (Zhou et al., 1992, 2015; Unwin and Bard, 1991). It has even recently been employed to evaluate the lifetime of intermediate species relevant to CO_2_ reduction (CO_2_^-.^ radicals in DMF) (Kai et al., 2017). While powerful, the need for reactions to be electrochemically reversible make SECM somewhat limited in terms of applicable reaction systems. Additionally, intricate probe fabrication and long experimental timescales severely limit achievable sample throughputs.

**Figure 6:**
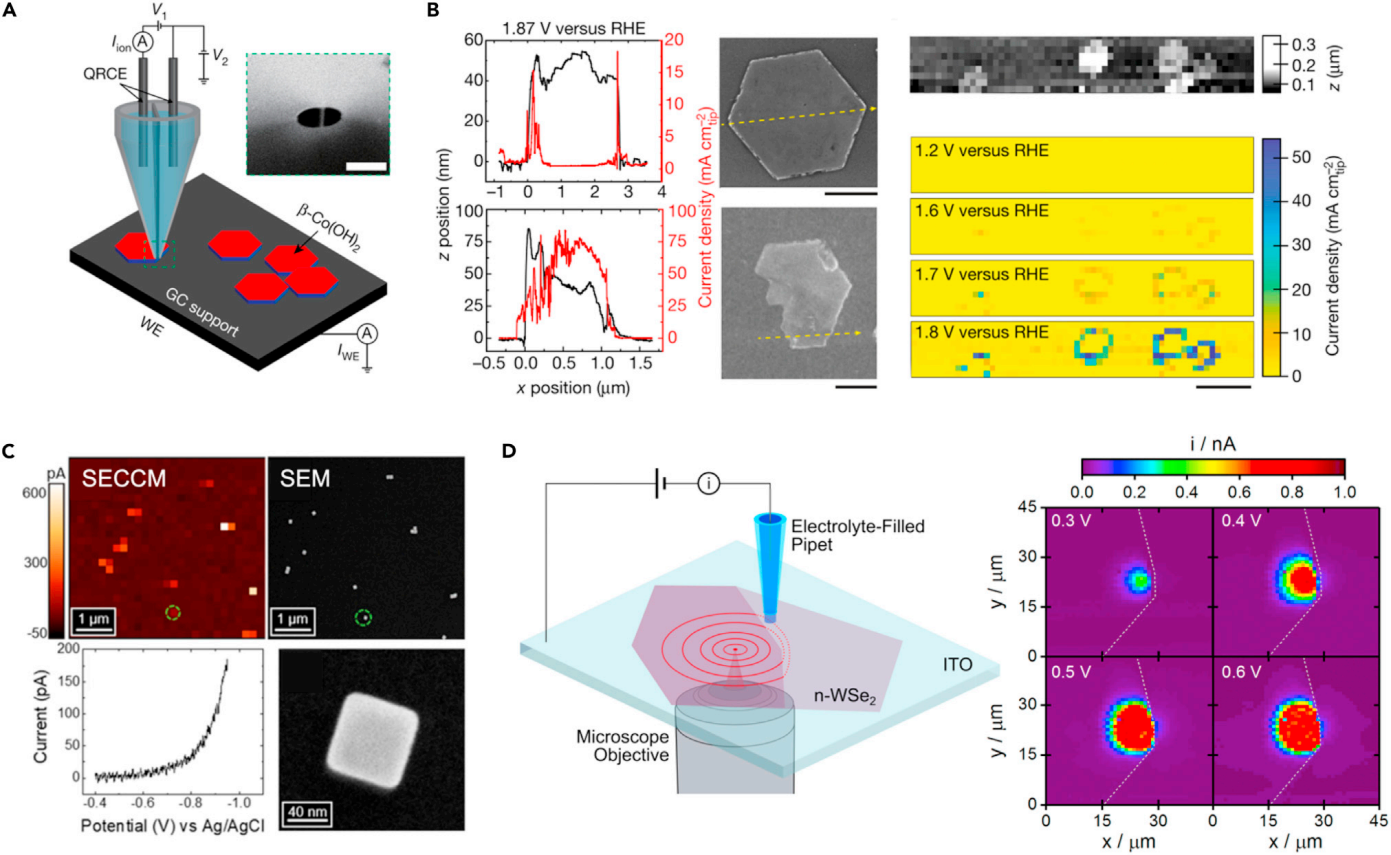
Locally mapping (photo)catalytic behavior using Scanning Electrochemical Cell Microscopy (A) General experimental schematic for scanning electrochemical cell microscopy (SECCM). A small pipet is filled with an electrolyte and a metal wire which serves as a quasi-reference counter electrode (QRCE). This probe is brought into contact with a sample which serves as a working electrode (WE), allowing direct voltammetric measurements to be carried out on small regions of a sample. (B) Oxygen evolution reaction (OER) catalysis at individual -Co(OH)_2_ particles supported on glassy carbon interrogated via SECCM. Left panel depicts topographic and OER current cross-sections generated via SECCM analysis of the picture particles. Right panel gives SECCM images of a sample region containing several Co(OH)_2_ particles. (C) Hydrogen evolution reaction (HER) catalysis at individual shape-controlled Au NPs on HOPG. Provided are an SECCM image of individual cubic Au NPs (−0.95 V vs. Ag/AgCl), an SEM image of the same sample region, a voltammogram recorded over an individual Au NP, and an SEM image of the same NP. (D) Carrier generation-tip collection SECCM. A localized excitation source is used to generate carriers locally at a point of interest on a sample. The transport of these carriers is visualized by measuring the rates of a reaction driven by these carriers at a spatially offset SECCM probe. The right panel depicts CG-TC SECCM imaging of iodide oxidation in the vicinity of a step-edge defect in n-WSe_2_. The electrolyte contained 0.1 M NaI, 0.01 M I_2_. All images adapted with permission from the indicated sources (Mefford et al., 2021; Choi et al., 2020; Hill and Hill, 2021).

In contrast to these approaches, which utilize a highly localized probe to interrogate an extended electrochemical interface, local information can also be generated by controlling how the electrochemical interface is defined. This philosophy is the basis of scanning electrochemical cell microscopy (SECCM), first demonstrated by Unwin et al. (Snowden et al., 2012; Lai et al., 2011; Kang et al., 2017; Güell et al., 2014; Bentley et al., 2019). In SECCM, a small, nm-scale electrolyte-filled pipet is brought into contact with a sample, creating a miniaturized electrochemical cell where a small region of the sample surface acts as a working electrode and a metal wire in the electrolyte acts as a counter electrode (Figure 6A). Voltammetry experiments can then be carried out which directly reflect the local chemical behavior of a sample. By carrying out a series of these measurements across an array of points on the sample, local variations in activity can be directly visualized.

SECCM holds several advantages over other techniques for locally interrogating the electrochemical properties of materials. The pipets employed as probes in SECCM are easily fabricated via laser-assisted pulling and can be prepared with terminal diameters approaching ∼10 nm. This enables spatial resolutions on the order of 50 nm to be easily achieved. Because the currents measured in SECCM directly reflect reaction rates at the sample surface, data interpretation and analysis are comparatively straightforward. A key drawback of SECCM, however, is the role electrolyte wetting plays in the spatial resolution of the technique. The exact geometry of the meniscus created upon probe-sample contact is often unknown and would depend on the surface energies of the electrolyte-sample, electrolyte-air, and electrolyte-pipet interfaces. Even so, a variety of strategies exist for minimizing the meniscus footprint, such as carrying out measurements under an immiscible oil phase (the strategy employed in the experiments depicted in Figure 6B). Precise footprint dimensions can be straightforwardly evaluated through ex situ characterization (e.g., electron microscopy of residual electrolyte) as well as *in situ* methods, such as interference reflection microscopy (Valavanis et al., 2021).

Figures 6B–6D illustrate some example applications of SECCM which demonstrate the power of this and related techniques for studying complex electrochemical systems (Mefford et al., 2021; Hill and Hill, 2021; Choi et al., 2020). Figure 6B shows the application of SECCM to interrogate the rates of the oxygen evolution reaction (OER) across μm-scale β-Co(OH)_2_ particles. Using SECCM, spatial variations in OER rates could be directly visualized across these platelet-like particles, and the authors find catalytic activity is highly localized along the particle edges. The authors ascribe this behavior to local changes in Co oxidation state within the particle, with an increase in Co^3+^ concentration driving the increased OER activity at the edge regions. Figure 6C depicts the application of SECCM to probe hydrogen evolution reaction (HER) catalysis at smaller, shape-controlled colloidal Au nanoparticles (NPs) (Choi et al., 2020). Owing to their smaller size (<100 nm), individual NPs were effectively trapped within the meniscus and SECCM measurements reflected the total catalytic rate across the NP surface. As a result, NPs appeared as isolated “pixels” in the SECCM images at expected locations based on correlated SEM imaging studies. In these studies, large NP-to-NP variations in kinetic behavior could be directly observed, ascribable to heterogeneities in ligand coverage between individual NPs. These variations were found to be greater for cubic NPs ({100}-capped) than octahedral NPs ({111}-capped). These SECCM approaches can be straightforwardly extended to visualize spatial heterogeneities in CO_2_RR activity across catalyst surfaces, serving as a powerful tool for identifying active structural motifs in the heterostructured catalyst materials considered here. SECCM studies of CO_2_ reduction catalysts have been limited thus far, but it has been successfully applied to map CO_2_RR activity across Au electrodes, where it was found that activity is enhanced locally at grain boundaries due to the accumulation of dislocations at these sites (Mariano et al., 2021; Mariano Ruperto et al., 2017).

In photocatalytic systems, the generation and transport of charge carriers which drive reactions will also play a critical role in the overall efficiency of a process. Figure 6D illustrates how SECCM can be applied to visualize the generation and transport of charge carriers in semiconducting materials (Hill and Hill, 2021). In this “carrier generation-tip collection” (CG-TC) mode, a localized excitation source, such as a focused laser, is utilized to generate carriers locally at a region of interest in a material. The transport of carriers can then be interrogated by measuring the rate of a photoelectrochemical reaction at the interface defined by a spatially displaced SECCM probe. By carrying a series of measurements with different excitation-pipet offsets, the transport of photogenerated carriers can be directly visualized. In the example depicted here, the transport of photogenerated holes in n-WSe_2_ was visualized by measuring the rate of iodide oxidation in the vicinity of a focused 633 nm laser. Using these CG-TC SECCM measurements, carrier diffusion lengths in n-WSe_2_ could be quantified and the influence of different types of defects on transport directly visualized. In the provided SECCM images, it can be seen that the presence of a single step-edge defect within an exfoliated WSe_2_ nanosheet completely limits hole transport due to carrier recombination.

## Virtuous cycle of experiments, spectroscopy, simulations, and data science

Owing to the sheer number of available combinations, it becomes impossible to perform experimental synthesis for heterostructured compounds one-by-one. Likewise, from a computational standpoint, not even the largest supercomputers would be able to calculate these structures in a reasonable timespan. Furthermore, the space of realizable materials is intrinsically high-dimensional, making traditional atomistic and quantum approaches inefficient for their study (Wei et al., 2019). Just to give an example, let's say we want to combine 2DMOF, Chevrel phases, and 2DMHPs to create a vdW heterostructure as highlighted in Figure 5. The number of possible combinations in heterostructures scales as ∼ *X*^*n*^, where *X* is the number of available single layer materials and *n* is the number of layers in the heterostructure. If we can create ∼800 types of 2D materials, the number of possible 8-layer heterostructures is ∼1 × 10^23^, almost as many as the stars in the observable universe. Likewise, the number of possible 27-layer heterostructures is ∼2 × 10^78^, comparable to the number of atoms in the known universe. Again, if this complexity arises by creating only 2D-2D heterostructures, imagine now when the other dimensionalities are included.

It is therefore evident that to try to solve this problem, synthesis, theory, simulations, and data science are to interact to complement each other. For example, experimental measurements can help validate theoretical models, while theory and simulations can help explain the different phenomena at different scales. From this perspective, this problem requires 1) a way to screen these compounds to determine the best combinations for heterostructure, and 2) algorithms to predict their final structure given different interactions to obtain design principles. One possible path is that data science and machine learning can help to scan experimental measurements faster or help the sharing of information between different measurements and theoretical models. This will generate a virtuous cycle, where the new machine learning models can make prediction beyond the training set or key parameters (dimensionality reduction), and experiments and theory can explore that combinatorics space (Fan et al., 2021; Ashwin Kishore et al., 2020). In the next cycle, the experiments and atomistic/quantum simulations can help improve the machine learning model and the cycle can continue. The advantage is that we do not have to explore all the parameter space, but rather the section that is optimal for CO_2_RR and light capture. This has the potential of accelerating discovery and improving our understanding of the different phenomena at different time and length scales.

## Conclusions and outlook

In this perspective, we have discussed multidimensional catalyst systems that are complex enough to help us shine light on how CO_2_ can be reduced selectively and efficiently. By modulating the dimensionality of materials such as Chevrel phases, metal chalcogenides, metal halide perovskites, and 2D metal-organic frameworks as well as the formation of vdW heterostructures, it is possible to increase the electro- and/or photocatalytic performance of these systems toward CO_2_ reduction. As the field moves forward, particularly in identifying efficient catalytic building blocks and hierarchical structures with complex surfaces suitable for CO_2_ conversion, it will also be critical to design precise characterization approaches capable of interrogating well-defined active sites under operating conditions. We thus believe that in order to reach to the 80%–100% carbon free emission target by 2050, it will be necessary to assemble a multidisciplinary team of experts that will take advantage of targeted and accelerated design of materials, operando spectroscopy and diffraction, theory, simulations, and data science. We also invite the field to think outside the box in terms of developing exotic hierarchical structures combining moieties of 0D, 1D, and/or 2D character as a mean to promote multiple functionalities such as efficient charge transfer, exciton dissociation, plasmon resonance, and uniquely active coordination sites. The complexity of these multi-variate problems is large, but so are the opportunities.
